# Comparison between thoracic paravertebral block and segmental thoracic spinal anesthesia in breast cancer surgery

**DOI:** 10.1186/s42077-022-00281-8

**Published:** 2022-12-05

**Authors:** Alaa Mazy, Ashraf El-Domiaty, Nabil Abdel Mageed, Abdel Aziz Motawi, Medhat Messeha

**Affiliations:** Department of Anesthesia and Surgical Intensive Care, Faculty of Medicine, Mansoura University, Mansoura, Egypt

**Keywords:** Thoracic paravertebral block, Segmental thoracic spinal anesthesia, Breast cancer, Mastectomy, Hemodynamics

## Abstract

**Background:**

Thoracic paravertebral block (TPVB) and segmental thoracic spinal anesthesia (STSA) can be used as sole anesthesia techniques alternative to general anesthesia for modified radical mastectomy in some critical patients. Both techniques were compared for efficacy and safety including detailed block characteristics, analgesia, patient’s and surgeon’s satisfaction, hemodynamics, respiration, and side effects.

**Results:**

Both techniques were successful, but fentanyl requirements were higher in TPVB group. The sensory loss was faster, wider, and longer in STSA group; however, it was associated with more hypotension. There was no motor block in the upper or lower limbs in TPVB group, while all patients in STSA group showed ipsilateral handgrip affection and to less extent wrists and then elbow flexion. While the ipsilateral lower limbs motor block was partial and short. Postoperatively, there was no difference in analgesic requirements or side effects. Satisfaction was higher in STSA group.

**Conclusions:**

Both TPVB and STSA were effective and safe as sole techniques for mastectomy providing adequate anesthesia with low complications, considerable analgesia, and satisfaction. Anesthesia was faster, wider, and longer in STSA group, with lower fentanyl requirements; however, it was associated with more hypotension.

## Background

Breast cancer is the most diagnosed and most lethal cancer among women globally (Winters et al. 2017). General anesthesia (GA) is commonly used for breast cancer surgery. However, in the time of COVID-19 (Días et al. 2021), and in some critical patients, regional anesthesia may be used as a sole technique including high thoracic epidural anesthesia (Yektas et al. 2014; Rangrez et al. 2020); combined facial plane blocks (Días et al. 2021; Gutiérrez et al. 2019; Munasinghe et al. 2021); paravertebral block (Buckenmaier et al. 2002; Oğuz et al. 2007; Nikam et al. 2014; Pangthipampai et al. 2020); segmental thoracic spinal anesthesia (Elakany and Abdelhamid 2013; Madishetti et al. 2017; Caruselli and Michel 2020); and tumescent anesthesia (Khater et al. 2017).

Thoracic paravertebral block (TPVB) provides safe anesthesia with balanced hemodynamic response through a unilateral somatic and sympathetic blockade, affords postoperative analgesia, early discharge, and low cost (Beyaz et al. 2012). Validating different anesthesia techniques against the most-established one of TPVB could guide the best choice (Chin et al. 2021). Thoracic spinal anesthesia (STSA) provides high quality of postoperative analgesia, shorter recovery time, and early hospital discharge (Elakany and Abdelhamid 2013). Nevertheless, no previous randomized prospective trials compared TPVB and STSA as regards block characteristics, efficacy, and safety in critical patients undergoing modified radical mastectomy.

## Methods

This randomized prospective study was done in Mansoura Oncology Center, after approval of the institutional review board (MD/15.05.91), and clinical trials registration number is NCT03319511.

Seventy-two female patients, undergoing unilateral modified radical mastectomy with axillary dissection and physical status American Society of Anesthesiologists (ASA) II-IV aged 35 to 70 years, were included. Patients with cardiovascular, pulmonary, renal, hepatic, and endocrinal diseases were accepted. Patients were excluded upon refusal for regional techniques or the presence of coagulopathy, local infection, and hypersensitivity to anesthetic drugs.

Complete medical history, clinical examination, and routine laboratory investigations were assessed (ECG, complete blood picture, coagulation profile, liver and renal function tests). Further investigations were done according to morbidity (as echocardiography, pulmonary function test). All patients were informed about the regional anesthesia techniques, and consent was written.

In the preparation area, an intravenous peripheral line was inserted opposite to surgical side. Preload with 10 ml/kg Ringer’s lactate was given except in patients with renal impairment, pulmonary congestion, or impaired systolic function. In the operating room, monitoring included electrocardiography (ECG), heart rate (HR), noninvasive mean blood pressure (MBP), and oxygen saturation (SpO_2_).

Patients were randomly assigned through the closed envelop method into two equal groups, the thoracic paravertebral block (TPVB) group and the segmental thoracic spinal anesthesia (STSA) group (each *n* = 35).

Regional procedures were done under complete aseptic precautions, and the skin infiltration was performed with 2 ml lignocaine 1% before needle puncture.

### Thoracic paravertebral block

Patients were in a setting position. Linear ultrasound (Siemens Acuason 300, Germany) probe of high-frequency linear transducer (7–12 MHz) was used to confirm the thoracic levels T2 and T4 that were marked at their superior aspect. The US probe was placed transversely, perpendicular to the longitudinal plane of the spinous processes. Medially, the transverse process was visualized, while the pleura appear under the inferolateral aspect. A 22 G spinal needle was introduced in-plane in a medial direction (Krediet et al. 2015), aiming to penetrate the internal intercostal membrane; after negative aspiration, 0.3 ml (1.5 mg)/kg of 0.5% plain bupivacaine in addition to 0.5 microgram (mcg)/kg dexmedetomidine (DEX) was slowly injected over 2–3 min. The dose was divided between two punctures at levels T2 and T4. Spread of local anesthetic leads to depression of the pleura.

### Segmental thoracic spinal anesthesia

The patient was in the sitting position with a flexed head. Determine the space and depth using ultrasound. In the parasagittal plane, 2 cm from the midline, the desired level (T5–6) was determined by an ultrasound (2–5 MHz) curved array probe through counting up from the last rib. A skin mark was placed to identify the correct level of the block. The probe is moved medially along the 5th rib echo to identify the ligamentum flavum and posterior dura hyperechoic lines (Salman et al. 2011).

A paramedian approach with a 25 G Quincke spinal needle was used. After piercing the ligamentum flavum, the needle’s stylet was removed, and the hub was observed for free flow clear cerebrospinal fluid (CSF); 1.5 ml of plain bupivacaine 0.5% in addition to 5 mcg DEX was injected. Then, the patient was placed in a lateral position on the surgical side for 15 min before shifting to a supine position.

The technique was abandoned upon patient request, if ultrasonic scanning of dura is not clear, more than 4 attempts, or repeated paresthesia. If paresthesia occurred, withdraw the needle 0.5–1 mm; the injection must be painless.

The onset of sensory block was evaluated by pinprick test with a 25-gauge needle along the anterior axillary line using a 3-point score: grade 0: Sharp pin felt; grade 1: analgesia, the dull sensation felt; and grade 2: anesthesia, no sensation felt. The block onset was tested every 5 min in TPVB group for 30 min, every 2 min in STSA group for 15 min after the completion of the injection, and then every 30 min following surgery until sensory regression.

Block success was defined as a complete sensory block in all T2–T6 dermatomes within 30 min of injection; analgesia and sedation — if required for painless surgery — were provided through increments of iv midazolam 1–2 mg for sedation, fentanyl 25 mcg for analgesia, and propofol 50 mg consecutively. General anesthesia (GA) was scheduled in case of block failure, compromised ventilation, difficulty to control pain, agitation, or upon patient request. Then, the case would be excluded from the study.

The motor block was evaluated before surgery; in the upper limbs, it was assessed by the epidural scoring scale for arm movements (ESSAM) score: handgrip (T1/C8), wrist flexion (C8/C7), and elbow flexion (C6/ C5); four grades (0–3) were based on the number of absent movements in the 3 joints ascendingly (Abd Elrazek et al. 1999). The motor block in the lower limbs was assessed by the modified Bromage scale (Cline et al. 2004). Motor regression in the limbs was assessed every 15 min until recovery.

Grading of sedation was evaluated by using the Ramsay sedation scale of 6 grades from 1: awake to 6: unarousable (Ramsay et al. 1974). The sedation score was recorded basal and then every 30 min during the procedure. Patients, postoperative assessors, and surgeons were blinded for procedure. The satisfaction was assessed after surgery for surgeons, while after 24 h for patients, using VAS (0–10), zero level was the least, and 10 was the highest satisfaction level.

### Postoperative assessment

Quality of analgesia was measured by visual analog score (VAS) on a 0–10 cm scale; 0 is no pain and 10 the maximum pain. VAS score was measured at 0, 2, 4, 6, 8, 12, 18, and 24 h postoperatively. Whenever VAS score is ≥ 4, analgesia was provided by oral paracetamol 1 g every 8 h, IV 30 mg of ketorolac every 8 h, and incremental doses of meperidine 20 mg as rescue analgesia if VAS is still ≥ 4. Total analgesic consumption in 24 h, number of patients requiring analgesia, and the time to first analgesic request were recorded. The incidence of complications was recorded including bradycardia, hypotension, nausea, vomiting, and hypoxia. Hypotension is considered on a 20% drop in baseline MBP or systolic pressure below 90 mmHg; it was treated with iv ephedrine 5 mg increments. Bradycardia is considered if HR ˂ 50 beat/min; it was treated with iv atropine 0.5 mg. Hypoxia is considered if oxygen saturation ˂ 90%; it was managed by oxygen mask, assisted breathing; if not sufficient for ventilation, a laryngeal mask or an endotracheal tube is inserted to proceed as GA.

### Statistical analysis

A pilot study including 5 patients in each group was conducted to determine the study sample size with regard to efficacy. The difference in fentanyl requirements was a differentiating outcome. The mean and standard deviation (SD) for TPVB group were 12 ± 9, while it was 5 ± 8 for STSA. Accordingly, the calculated effect size was 0.822 using the priory G*Power two-tailed test. Assuming *α* error of 0.05, and a power of 90%, the sample size was 66. An additional 5% was considered for data drop, so the sample size was 70 divided into 35 for each group. The results of this pilot study are also implemented increasing the spinal dose from 1 to 1.5 ml of bupivacaine for better coverage of breast dermatomes. Also, adopting the lateral position for 15 min after spinal injection thus increases block selectivity and safety.

The collected data were coded, processed, and analyzed using SPSS (Statistical Package for Social Science, SPSS Inc., Chicago, IL, USA) program version 21 for windows. Shapiro-Wilk test was used to check the normality of data distribution. Qualitative data were described using numbers and percent. Association between categorical variables was tested using the chi-square test or Fisher exact test. Continuous variables were presented as mean ± SD for parametric data and median (min-max) for nonparametric data. The two groups were compared with the Student *t*-test for parametric data, while the Mann-Whitney test was used to compare nonparametric data.

## Results

**Fig. 1:**
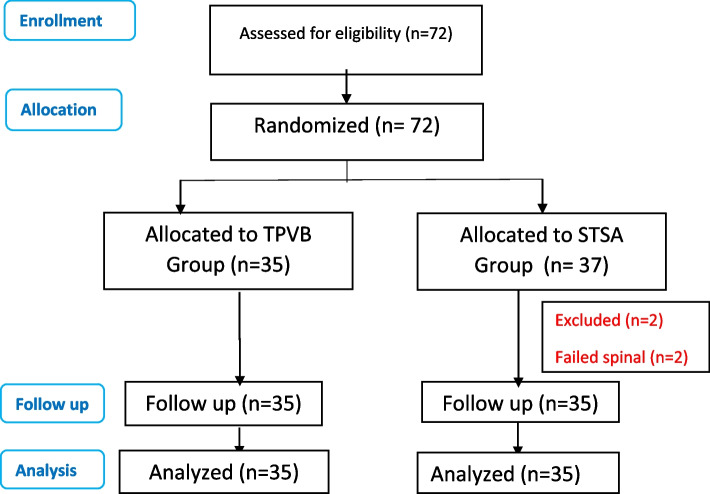
Consort flow diagram

Both groups were comparable for demographic and surgical data (Table 1). Many patients were afraid of regional anesthesia, but they accepted after explanation of the techniques and its safety, especially they had some medical compromise (Table 2), in addition to the ability to revert to GA if required. Seventy-two patients were included; only two were excluded due to difficult spinal (Fig. 1).

**Table 1:** Demographic data

Items	TPVB group	STSA group	***p***-value
Age (years)	52.7 ± 11.0	52.0 ± 9.5	0.753
BMI (kg/m^2^)	32.7 ± 3.0	31.3 ± 3.6	0.091
ASA physical status			0.257
II	18 (51.4%)	20 (57.1%)	
III	16 (45.7%)	14 (40%)	
IV	1 (2.9%)	1 (2.9%)	
Surgery duration (min)	74.5 ± 10.5	73.5 ± 11.6	0.722

Data are expressed as mean ± SD or number and percentage (%), *n* = 35

**Table 2:** Detailed coexisting diseases

Items	TPVB group (***n*** = 35)	STSA group (***n*** = 35)	***p***-value
Diabetes mellitus	16 (45.7%)	16 (45.7%)	1
Bronchial asthma	5 (14.3%)	4 (11.4%)	0.732
Hypertension	23 (65.7%)	24 (68.6%)	0.801
Hepatitis	5 (14.3%)	6 (17.1%)	0.744
Ischemic heart disease	3 (8.6%)	0 (0.0%)	0.079
Mitral regurge	2 (5.7%)	1 (2.9%)	0.558
Hyperthyroidism	2 (5.7%)	0 (0.0%)	0.154
Hypothyroidism	0 (0.0%)	1 (2.9%)	0.317
Respiratory failure	1 (2.9%)	0 (0.0%)	0.317
Rheumatoid	0 (0.0%)	1 (2.9%)	0.317
Multiple disease	18 (51.4%)	17 (48.6%)	0.811

*Data are expressed* as number (*n*) and percentage (%)

No cases required GA, but the intraoperative fentanyl requirements were higher in TPVB group (Table 5).

The onset of sensory block was faster, and its extent was wider, and the duration of the block was longer in STSA group (Table 3). There was sufficient anesthesia in the ipsilateral side in both groups. Sixteen patients (45.7%) felt a dull sensation in the contralateral side of surgery within 10 min after spinal injection. Eight patients (22.9%) in STSA group complained of dyspnea within 10 min of spinal injection that was ameliorated by an oxygen mask holding a positive expiratory valve at 10 cmH_2_O and reassurance; SpO_2_ was 100%.

**Table 3:** Sensory block data

Items	TPVB group	STSA group	***p***-value
Onset of sensory block (min)	20.2 ± 3.0	6.4 ± 1.4	< 0.001*
Number of blocked dermatomes	8 (7–10)	10 (7–16)	< 0.001*
Sensory regression time (min)	164 ± 18	175 ± 12	0.004*

Data are expressed as mean ± SD, median (range), *n* = 35. *Statistically significant *p* ≤ 0.05

There was no motor block in the upper or lower limbs in TPVB group. However, there was a significant mild ipsilateral limb blockade in STSA group; the block was longer in the upper than lower limbs (Table 4).

**Table 4:** Detailed motor block characteristics in the upper limbs (ESSAM score) and lower limbs (modified Bromage score) in both groups

**Upper limbs**	**Ipsilateral**			**Contralateral**		
**ESSAM score**	**TPVB**	**STSA**	***p*-value**	**TPVB**	**STSA**	***p*-value**
**0**	**35 (100%)**	**-**		**35 (100%)**	**32 (91.4%)**	
**1**	**-**	**35 (100%)**	< 0.001*	**-**	**3 (8.6%)**	**0.079**
**2**	**-**	**25 (71.4%)**	< 0.001*	**-**	**-**	
**3**	**-**	**5 (14.3%)**	0.021*	**-**	**-**	
**Duration of block (min)**	**0**	**26.62 ± 4.31**				
**Lower limbs**	**Ipsilateral**			**Contralateral**		
**Modified Bromage scale**	**TPVB**	**STSA**	***p*-value**	**TPVB**	**STSA**	***p*-value**
**0**	**35 (100%)**	**16 (45.7%)**	< .001*	**35 (100%)**	**31 (88.6%)**	0.039*
**1**	-	**15 (42.9%)**		-	**4 (11.4%)**	
**2**	-	**4 (11.4%)**		-	-	
**3**	-	-		-	-	
**Duration of block (min)**	**0**	**13.45 ± 2.32**				

Data are in number and percentage (%). *n* = 35. *Statistically significant *p* ≤ 0.05. The epidural scoring scale for arm movements (ESSAM) score: elbow flexion (C6/ C5); wrist flexion (C8/C7); handgrip (T1/C8). Grades (0, no block; 1, no hand grip; 2, no hand or wrist flexion; 3, no hand, wrist, or elbow flexion). The grade of modified Bromage scale (0, free movement of legs and feet; 1, just able to flex knees with free movement of feet; 2, unable to flex knees but free movement of feet; 3, unable to move legs or feet)

**Fig. 2:**
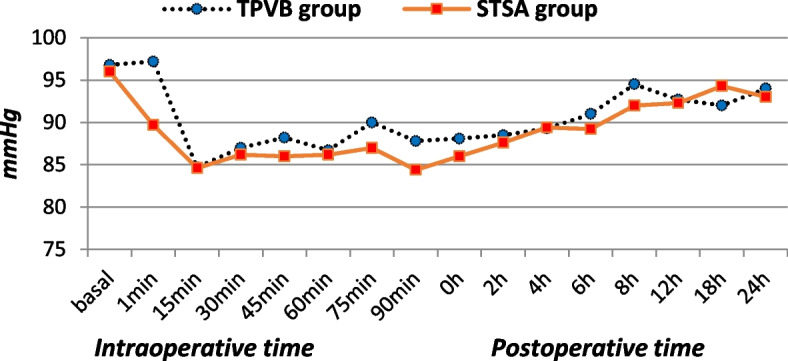
Perioperative mean blood pressure (mmHg) of the two studied group

**Fig. 3:**
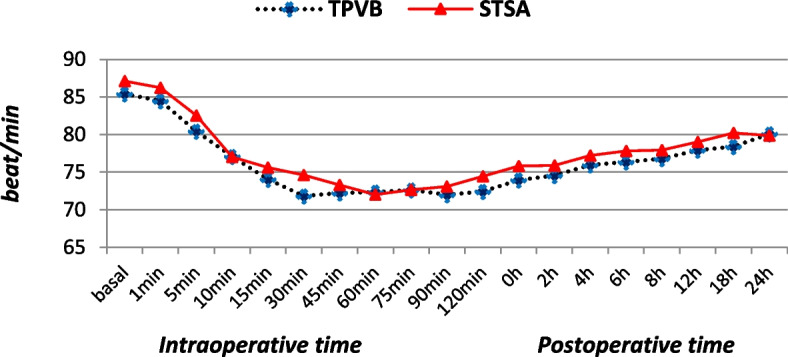
Perioperative heart rate (beat/min) of the two studied groups

There was no significant perioperative difference as regards MAP (Fig. 2), HR (Fig. 3), or oxygen saturation (mostly around 98%). However, the number of patients who developed hypotension and subsequent ephedrine utilization was significantly higher in STSA group; the maximum dose of ephedrine was 20 mg (Table 5).

**Table 5:** The intraoperative anesthetic drug requirements: incidences and doses

Drug	TPVB group	STSA group	***p***-value
Midazolam *n* (%)	11 (31.4%)	8 (22.9%)	0.420
Midazolam dose (mg)	0 (0–2)	0 (0–5)	0.365
Fentanyl *n* (%)	13 (37.1%)*	5 (14.3%)	0.029
Fentanyl dose (mcg)	0 (0–50)*	0 (0–25)	0.015
Propofol *n* (%)	5 (14.3%)	4 (11.4%)	0.721
Propofol dose (mg)	0 (0–100)	0 (0–100)	0.641
Ephedrine *n* (%)	12 (34.3%)	24 (68.6%)*	0.004
Ephedrine dose (mg)	0 (0–15)	0 (0–20)*	0.028
Atropine *n* (%)	3 (8.6%)	3 (8.6%)	1
Atropine dose (mg)	0 (0–0.4)	0 (0–1)	0.112

*Data expressed* as number (*n*) and percentage (%) or median (range). *n* = 35, *significant *p* ≤ 0.05

The incidence of intraoperative sedation was comparable in both groups (Table 6). Also, the timed Ramsay sedation scale was not different between the groups (Table 7).

**Table 6:** Intraoperative sedation data of the two groups

Patients (***n*** & %)	TPVB group	STSA group	***p***-value
Not sedated (*n* & %)	12 (34.3%)	14 (40%)	0.744
Sedated with drugs (*n* & %)	13 (37.1%)	10 (28.6%)	
Sedated without drugs (*n* & %)	10 (28.6%)	11 (31.4%)	

Data are expressed in number and percent (*n* = 35)

**Table 7:** Intraoperative Ramsay sedation score of the two groups

Ramsay sedation score	TPVB group	STSA group	***p***-value
**After 30 min**			0.08
1 = awake	12 (34.3%)	19 (54.3%)	
2 = calm	12 (34.3%)	7 (20.0%)	
3 = awake on verbal command	8 (22.9%)	9 (25.7%)	
4 = respond to tactile stimulus	3 (8.6%)	0 (0%)	
**After 60 min**			0.1
1 = awake	20 (57.1%)	26 (74.3%)	
2 = calm	13 (37.1%)	9 (25.7%)	
3 = awake on verbal command	2 (5.7%)	0 (0%)	
**After 90 min**			0.43
1 = awake	30 (85.7%)	28 (80%)	
2= calm	5 (14.3%)	7 (20%)	

Data are expressed in number and percent (*n* = 35). Ramsay sedation score: 1, awake, conscious, no sedation; 2, calm and compose; 3, awake on verbal command; 4, brisk response to gentle tactile stimulation; 5, awake on vigorous shaking; 6, unarousable

The incidence of intra- and postoperative complications was not different except for more hypotension in STSA group (Table 8). Only 2 patients (5.7%) in STSA group developed a respiratory compromise (*SpO*_*2*_ < 90%) mostly associated with hypotension; they were managed with supplemental oxygen and control of blood pressure. There were no other block-related complications as vascular puncture, paresthesia, or pneumothorax. Postoperatively (Table 8), only 2 cases developed postoperative nausea and vomiting in STSA group during events of hypotension that resolved after the correction of hypotension with no need for an antiemetic. There was no hypoxia, bradycardia, urine retention, or headache.

**Table 8:** The incidence of intra- and postoperative complications in number (percent)

Complications	Operative time	TPVB group	STSA group	***p***-value
Hypotension	Intra	9 (25.7%)	22 (62.9%)*	0.002*
	Post	1 (2.9%)	3 (8.6%)	
Bradycardia	Intra	2 (5.7%)	3 (8.6%)	0.645
	Post	0 (0%)	0 (0%)	
Hypoxia	Intra	0 (0%)	2 (5.7%)	0.154
	Post	0 (0%)	0 (0%)	
Nausea	Intra	0 (0%)	0 (0%)	1
	Post	0 (0%)	1 (2.9%)	
Vomiting	Intra	0 (0%)	0 (0%)	1
	Post	0 (0%)	1 (2.9%	

Data are in number and percentage (%). *n* = 35. *Statistically significant *p* ≤ 0.05. Hypoxia (*SpO2* < 90%), hypotension (20% drop in baseline MBP or systolic pressure < 90 mm Hg), bradycardia (*HR* < 50 beat/min)

**Fig. 4:**
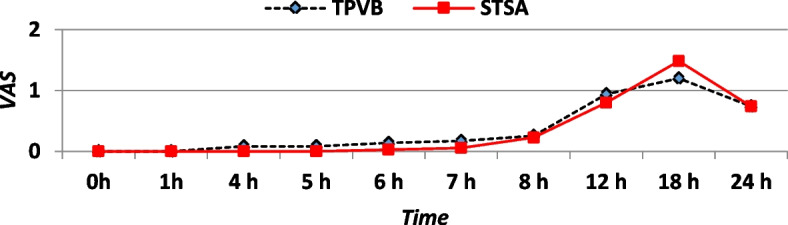
The mean postoperative visual analog scores for pain (VAS)

The duration to the 1st request for analgesia was longer in STSA group, and analgesic consumption (ketorolac) during the first 24-h postoperative was comparable in both groups (Table 9). There were no postoperative opioid requirements in both groups. The median values of the VAS score were not different between the groups during the first 24 h (Fig. 4). Patient and surgeon satisfaction was higher in STSA groups (Table 9).

**Table 9:** Data of postoperative analgesia and satisfaction for patients and surgeons

Item	TPVB group	STSA group	***p***-value
Duration to 1st analgesic request (min)	855 ± 232	993 ± 218	0.012*
Ketorolac consumption in 24 h (mg)	46.3 ± 25	37.7 ± 17	0.093
Surgeon satisfaction (score 0–10)	8.7 ± 0.8	9.3 ± 1	0.007*
Patient satisfaction (score 0–10)	9.1 ± 1.0	9.6 ± 0.8	0.011*

Data are expressed in mean and standard deviation. *n* = 35. *Statistically significant *p* ≤ 0.05

## Discussion

This study compared TPVB and STSA during modified radical mastectomy in some critical patients. Precise knowledge of detailed block characteristics and safety profiles is essential in this patient category. Both techniques were efficient; no cases mandated GA, low-dose requirements of intraoperative sedatives and analgesics, in addition to good postoperative analgesia. Effectively, STSA showed the advantage of clear endpoint (CSF), low-dose bupivacaine and DEX, rapid onset, wider sensory block, lower fentanyl requirements, and longer analgesia.

The lower requirements of fentanyl in STSA group may reflect the dense sensory, motor, and sympathetic influence of intrathecal block (Kowalewski et al. 2011). In addition, there was more dermatomal distribution in STSA group. The ipsilateral arm block involved all patients at handgrip level (T1-C8), 71.4% at wrist level (C8-C7), and 14.3% only at elbow level (C6-C5). This partial arm block implicates brachial plexus involvement that shares intercostal nerves for breast and axillary innervation (Seidel et al. 2017) and also eliminates phrenic nerve involvement (C3, 4, 5). Limited pectoral fascia block manifests TPVB (Pangthipampai et al. 2020). Ahmed et al. found upper limb block in 16% only of patients (Ahmed et al. 2014). The higher percent in our patients may be explained by higher bupivacaine dose (1.5 ml), the lateral position, and DEX block potentiation.

Sensory block extended about 3 h (163.65 min in TPVB, compared to 174.65 in STSA group). Ahmed et al. reported a slightly shorter duration, 157min (140–190) without DEX (Ahmed et al. 2014).

According to 1st analgesic request time, we report a duration of 16–19 h that was longer in STSA group. Only 10% of patients requested analgesia after abdominal surgery under STSA (Ellakany 2014). Al Mostafa et al. showed a time of 11.2 ± 1.5h after TPVB using 20 ml bupivacaine 0.25% at the T4 level (Moustafa et al. 2020). A meta-analysis by Wang et al. reported 200 min extra by adding DEX 1 mcg/kg in TPVB (Wang et al. 2018). The long duration of analgesia in our groups may be related to DEX, in addition to preventive analgesia through reducing central sensitization and avoiding opioid hyperalgesia (Gayraud et al. 2020). Therefore, pain is halved for 3–6 months after surgery using PVB (Qian et al. 2019). The long duration of analgesia and low VAS in both groups implement opioid-free analgesia with its concomitant benefits. Despite the controversy, TPVB may reduce immunosuppression and cancer metastasis (Kulkarni 2016). In this study, about 30% of patients were sedated without additional drugs in both groups that may be due to DEX.

We compared three safety items: hemodynamics, respiratory, and side effects. Primarily, STSA is more than a century-old technique (Imbelloni 2011). Magnetic resonance image (MRI) studies showed wide T5 space (7.75 mm) between the dura and the spinal cord, in addition to the longer hypotenuse from the point of entry to the cord due to an angle of almost 50° at the thoracic level (Imbelloni et al. 2010). In this study, there was no paresthesia in either group. That was in agreement with other studies (Elakany and Abdelhamid 2013), (El Moutaz et al. 2018). Van Zundert et al. reported an incidence of 5% paresthesia but during combined spinal-epidural (Tuohy) needle insertion. It was relieved after slight needle withdrawal without sequelae (Van Zundert et al. 2007). The consensus is that STSA is associated with a low incidence of hypotension and no neurologic problem (Imbelloni and Gouveia 2016).

Hemodynamically, both groups were comparable for bradycardia; only 3 patients (8.6%) in each group required atropine. Despite the efficacy of STSA, its safety may be compromised by more hypotension (25.7 vs. 62.9%). About two-thirds of patients required ephedrine, but it was easily treated within a range of 5–20 mg. We may explain the relative hypotension in STSA group by associated lumbar sympathetic block (concomitant ipsilateral lower limb weakness) in about half of patients (54.3%) but only for a short time (13.5 min). In contrast, there was no motor blockade in Ahmed et al. study. However, they found a 16% incidence of hypotension in ASA I patients (Ahmed et al. 2014). A similar incidence (15%) occurred in ASA I-III patients, but it was more frequent (20%) with GA (Elakany and Abdelhamid 2013). Vasoplegia also may be related to thoracic sympathetic blockade and subsequent cardiac sympathectomy. However, the acute hemodynamic changes during complete bilateral cardiac sympathetic denervation (stellate, T2-4 ganglia) under GA were significant but not serious; systolic BP decreased 14 mmHg, while HR decreased 20 b/min (81 to 61) (Sinkar et al. 2020).

In this study, TPVB group showed no upper or lower limb blockade. This high selectivity may reflect hemodynamic stability. In TPVB group, hypotension occurred in one-quarter of patients. The sympathetic blockade, DEX, and epidural spread may provide explanations. Kulkarni found no bradycardia, hypotension, or desaturation with TPVB during mastectomy while using DEX (Kulkarni 2016). However, in a multilevel TPVB under DEX infusion, there was hypotension in 32% of patients (Pangthipampai et al. 2020).

Consecutively, STSA risk for cardiac patients may not be clear in this study. Therefore, investigating a larger number and diversity of these patients is recommended. However, STSA may not be risky in cardiac patients (Park and Lee 2017). Cardiac sympathectomy is antiarrhythmic and antifibrillatory in high-risk patients for ventricular fibrillation (Schwartz 2014). The positive oxygen balance through coronary dilatation and decreased myocardial work is suitable with ischemic cardiomyopathy. Also, it is associated with predominant vagal activity that carries a potential benefit in patients with heart failure (Wu and Vaseghi 2020). Also protect against the down-regulation of β receptors (Lee et al. 2003). Kowalewski et al. omitted the perception that hypotension or bradycardia is dangerous with cardiac sympathectomy, where treatment is easy by proper vasoactive drugs. They preferred high spinal anesthesia in cardiac patients even with aortic stenosis, ischemia, or compromised myocardium (Kowalewski et al. 2011).

DEX — as an adjuvant — may endorse more hypotension, but it is transient and easily controlled by ephedrine. However, it reduces pain and prolongs analgesia (Wang et al. 2018). Meanwhile, DEX preconditioning provides cardiac protection through reduced myocardial injury and inflammatory stress response in cardiac surgery (Chen et al. 2021).

As regards respiratory safety, oxygen saturation is maintained in both groups that can be explained by ipsilateral intercostal selectivity and phrenic nerve sparing (C3, 4, 5). Only 2 patients (5.7%) in STSA group suffered from hypoxia that was associated with hypotension. Many studies found no signs of respiratory compromise (like apnea, hypopnea, or hypoxemia) in any patients under STSA anesthesia (Elakany and Abdelhamid 2013; Ahmed et al. 2014; Ellakany 2014; Kulkarni 2016). Therefore, STSA was interesting in patients with respiratory diseases as COPD (Caruselli and Michel 2020). In TPVB, 0.4 ml/kg bupivacaine at T4 level produced no clinically significant impairment of pulmonary function; FEV1 decreased from 1.97 to 1.7, and PEF from 4.4 L/s to 3.8, *P* < 0.01, but these effects may be related to associated fentanyl and midazolam sedation (Hura et al. 2017).

There was a low incidence of postoperative nausea and vomiting (PONV) in both groups. Only 1 case (2.8%) developed PONV in STSA group during an event of hypotension. Low incidence (10%) of PONV in STSA group was also confirmed (Elakany and Abdelhamid 2013). There were no cases with pneumothorax in this study. Also, Pace et al. reported 0% pneumothoraxes in 1427 ultrasound-guided TPVB (Pace et al. 2016).

## Conclusions

Both TPVB and STSA were effective sole techniques for mastectomy and axillary clearance providing adequate anesthesia with low complications, considerable analgesia, and high satisfaction. Anesthesia was faster, wider, and longer with lower fentanyl requirements in STSA group; however, it was associated with more hypotension. To confirm safety, a larger number and variety of medically compromised patients are recommended.

## Data Availability

The analyzed data are included in the tables. The details are available from the corresponding author upon a reasonable request.

## Abbreviations

TPVB: Thoracic paravertebral block
STSA: Segmental thoracic spinal anesthesia
ASA: American Society of Anesthesiologists
ECG: Electrocardiography
HR: Heart rate
MBP: Mean blood pressure
SpO2: Oxygen saturation
GA: General anesthesia
mcg: Microgram
DEX: Dexmedetomidine
US: Ultrasound
ESSAM: Epidural scoring scale for arm movements score
VAS: Visual analog score
CSF: Cerebrospinal fluid
MRI: Magnetic resonance imaging
PONV: Postoperative nausea and vomiting
